# Theoretical Demonstration of the Interest of Using Porous Germanium to Fabricate Multilayer Vertical Optical Structures for the Detection of SF_6_ Gas in the Mid-Infrared

**DOI:** 10.3390/s22030844

**Published:** 2022-01-22

**Authors:** Rami Zegadi, Nathalie Lorrain, Sofiane Meziani, Yannick Dumeige, Loїc Bodiou, Mohammed Guendouz, Abdelouahab Zegadi, Joël Charrier

**Affiliations:** 1 Institut FOTON-UMR 6082, CNRS, University of Rennes 1, F-22305 Lannion, France; nathalie.lorrain@univ-rennes1.fr (N.L.); sofiane.meziani@univ-rennes1.fr (S.M.); yannick.dumeige@univ-rennes1.fr (Y.D.); loic.bodiou@univ-rennes1.fr (L.B.); mohammed.guendouz@univ-rennes1.fr (M.G.); joel.charrier@univ-rennes1.fr (J.C.); 2 LEPCI Laboratory, Department of Electronics, Faculty of Technology, Ferhat Abbas University Sétif 1, Sétif 19000, Algeria; abdelouahabzegadi@univ-setif.dz

**Keywords:** porous germanium materials, mid-infrared detection, Bragg reflector, optical microcavity

## Abstract

Porous germanium is a promising material for sensing applications in the mid-infrared wavelength range due to its biocompatibility, large internal surface area, open pores network and widely tunable refractive index, as well as its large spectral transparency window ranging from 2 to 15 μm. Multilayers, such as Bragg reflectors and microcavities, based on porous germanium material, are designed and their optical spectra are simulated to enable SF_6_ gas-sensing applications at a wavelength of 10.55 µm, which corresponds to its major absorption line. The impact of both the number of successive layers and their respective porosity on the multilayer structures reflectance spectrum is investigated while favoring low layer thicknesses and thus the ease of multilayers manufacturing. The suitability of these microcavities for mid-infrared SF_6_ gas sensing is then numerically assessed. Using an asymmetrical microcavity porous structure, a sensitivity of 0.01%/ppm and a limit of detection (LOD) around 1 ppb for the SF_6_ gas detection are calculated. Thanks to both the porous nature allowing gases to easily infiltrate the overall structure and Ge mid-infrared optical properties, a theoretical detection limit nearly 1000 times lower than the current state of the art is simulated.

## 1. Introduction

Germanium (Ge) is a very promising material for spectroscopy and sensing applications in the mid-infrared (Mid-IR) wavelength range. Ge is advantageous because of its special physico-chemical properties [1,2]. In particular, its large spectral transparency window ranging from 2 to 15 μm, covering the whole molecule fingerprint, and its high refractive index allows the implementation of devices with reduced footprint. In recent years, numerous Ge-based fundamental devices have been developed using Ge-on-silicon [3,4], Ge-on-silicon-on-insulator [5], Ge-on-Si_3_N_4_ [6], and germanium-on-insulator [7] platforms.

Porous materials are attractive materials for many different sensing applications because of their large internal surface area [8,9], open pores network [10], and widely tunable refractive index [11]. Several structures using porous materials, especially porous silicon (PSi), have been demonstrated, such as omnidirectional mirrors [12], multilayers [13], microcavities [14], and waveguides [15]. The potential application areas of porous materials are mainly in the fields of biotechnology [16], microelectronics [17], and energy conversion [18]. These porous materials are promising candidates for environmental monitoring applications as they enable increased interaction between the wave and the molecules to be detected, which enhances sensor performance [19,20,21]. Porous silicon (PSi) sensing devices have been demonstrated to display greater sensitivities and lower limits of detection compared to massive materials in the near infrared (NIR) wavelength range [22,23]. Compared to silicon (Si), whose transparency window ranges from 1 to 8 μm, porous Ge (PGe) benefits from the larger transparency window of Ge extending from 2 to 15 μm. Furthermore, the strong light–matter interaction, conferred by its pores network, has attracted a growing interest for its use in various integrated detection applications [15,24].

This work is a contribution to the spectroscopic sensing of sulfur hexafluoride (SF_6_) gas whose Mid-IR signature monitoring could enable the quantification of its releases in the atmosphere [25]. This gas is mainly released by the electrical industry, which uses it as a gaseous insulator, and its concentration is rapidly increasing in the atmosphere. It is a powerful greenhouse gas listed in the Kyoto Protocol [26]. In its gaseous form, SF_6_ has a main absorption peak in the mid-infrared at 10.55 μm [27]. Using 2D material-based sensors, several techniques have been applied to SF_6_ gas detection: absorption of Ru-doped MoS_2_ (Ru-MoS_2_) [28], detection and absorption of InN doped with Ru (Ru-InN) [29], and detection Ni-doped C_3_N (Ni-C_3_N) [30]. An Ni-modified carbon nanotube (Ni-CNT) gas sensor was also implemented for SF_6_ detection and demonstrated a 1 ppm experimental limit of detection [31].

Leveraging from PGe advantages for Mid-IR sensing applications, the aim of the paper is to numerically study vertical optical structures based on PGe such as Bragg reflectors and microcavities dedicated to SF_6_ gas sensing. The study is first carried out on Bragg mirrors by varying both the number of layers N and the contrast between the low and high porosities of these layers to achieve a maximum reflectance R. Then, the study is focused on microcavities, which are the superposition of Bragg mirrors already studied, in order to define the numbers and porosities of the layers to get an optimal microcavity spectral response. In particular, the use of asymmetric microcavities is studied. Finally, a SF_6_ gas sensing study is performed based on the designed microcavities. Theoretical sensitivity and limit of detection are finally calculated and compared to the state of the art.

## 2. Modeling

The optical response of porous layers and their sensitivity to SF_6_ absorption strongly depend on the properties of a single layer (thickness, refractive index related to porosity, and pores size), and on the chosen optical multilayer structure (Bragg reflector or microcavity) fabricated using a stack of two different porous layers. The transfer matrix method was used to calculate the reflectance spectrum and to study the optical response as a function of the porous layer physical parameters (porosity, thickness, and number of layers) [32].

The refractive index of each layer is a key element to obtain the reflectance spectrum of the multilayered structures. A PGe layer of porosity *p* consists of germanium crystallites and open pores into which air infiltrates. The SF_6_ present in the air will therefore also penetrate the porous layer. Consequently, the complex refractive index of the PGe layer depends on its porosity *p* and on the superstrate consisting of air and SF_6_ with a concentration *C*. The presence of SF_6_ just influences the imaginary part *k* of the complex refractive index of the superstrate. In the presence of gas, *n*_sup_, which is the superstrate refractive index, is written in the form:

(1) $$n_{\sup}=n_{air}-jk,$$

with $k=\frac{\varepsilon (\lambda )\lambda }{4\pi }$, where λ is the wavelength, and *ε*(*λ*) is the absorption coefficient of SF_6_ in air.

The absorbance of SF_6_ in the Mid-IR wavelength range for different SF_6_ concentrations was extracted from the Hitran database [33]. The mid-IR complex refractive index of Ge, *n_Ge_*, was taken from [34,35].

The PGe refractive index, n, calculation was then performed, as a function of the PGe porosity and for different SF_6_ concentrations, using the Bruggeman model [36]:

(2) $$(1-p)\frac{n_{Ge}{}^{2}-n^{2}}{n_{Ge}{}^{2}+2n^{2}}+p\frac{n_{\sup}-n^{2}}{n_{\sup}+2n^{2}}=0,$$

**Figure 1:**
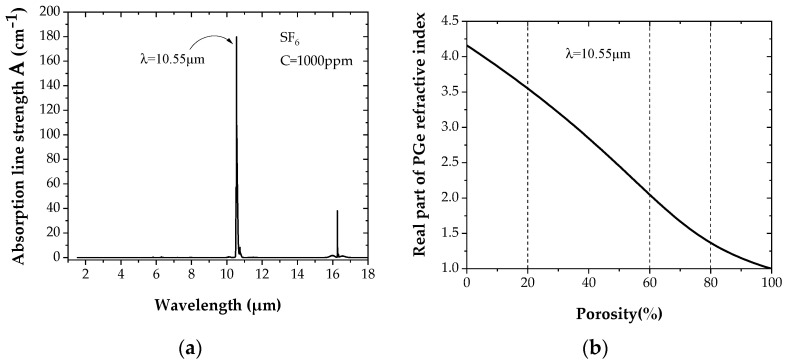
(**a**) Mid-IR absorbance spectrum of SF_6_ (for a SF_6_ concentration of 1000 ppm in air). (**b**) Dependence of the real part of the PGe refractive index on porosity.

The absorbance (A = C*ε*) spectrum for a SF_6_ concentration in air of C = 1000 ppm [37] is reported in Figure 1a. A strong SF_6_ absorption peak at a wavelength of 10.55 μm is observed. The transparency of germanium for such wavelengths is expected, hence validating its selection instead of silicon.

Figure 1b reports the strong dependence on the real part of n, the PGe refractive index on layer porosity at 10.55 μm, which allows multilayers with a high refractive index contrast to be obtained thereafter. The refractive index *n* obviously decreases with porosity. By selecting a given degree of porosity, a precise corresponding refractive index value n can be obtained from Figure 1b.

### 2.1. Bragg Reflector Theory

Bragg reflectors consist of periodic dielectric layers, with a quarter wavelength-optical path length for each layer giving them important properties and making them suitable for optoelectronics applications such as filters or laser cavity. It consists of a stack of alternating thin dielectric layers with high and low refractive indices. The reflectivity of the mirror is characterized by a stop band of high reflectivity. The pattern is formed by two layers of high and low porosities (named HP and LP, respectively) which is repeated N times. Each layer is characterized by its thickness *e* and its porosity, i.e., its refractive index. The Bragg reflector is characterized by its central wavelength λ_0_ (at normal incidence) and by the reflection bandwidth Δλ which is determined mainly by the index contrast. These two parameters are defined, respectively, by Equations (3) and (4).

(3) $$\lambda_{0}=2\left(n_{LP}e_{LP}+n_{HP}e_{HP}\right),$$

(4) $$\Delta \lambda =\frac{2\lambda_{0}\Delta n_{B}}{\pi n},$$

(5) $$\Delta n_{B}=n_{LP}-n_{HP},$$

where *n_HP_* and *e_HP_* are, respectively, the refractive index and the thickness for the HP layer, *n_LP_* and *e_LP_* for the LP layer, and $n_{B}=\frac{n_{LP}+n_{HP}}{2}$.

**Figure 2:**
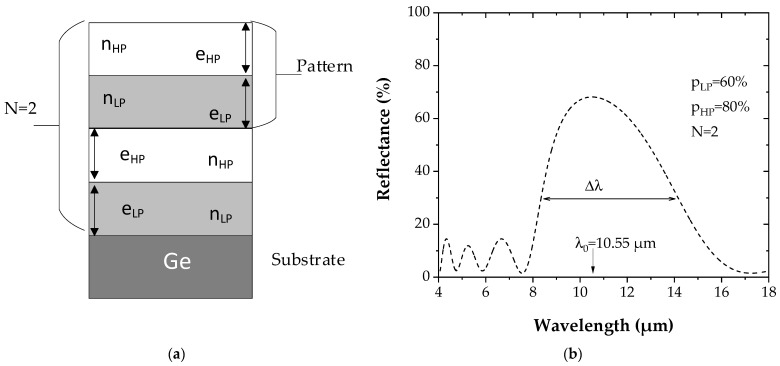
(**a**) Schematic of a PGe Bragg reflector made of a repeated pattern constituted by alternating a layer of low porosity (refractive index *n_LP_* and thickness *e_LP_*) and a layer of high porosity (refractive index *n_HP_* and thickness *e_HP_*). In this case, the pattern is repeated twice (N = 2). (**b**) Calculated reflectance spectrum of the corresponding PGe Bragg reflector, with values of 60% and 80% for the low and the high porosities, respectively.

Figure 2a represents a schematic Bragg reflector with a pattern composed of low and high porosity successive layers and repeated twice (N = 2).

The reflectance *R* of a Bragg mirror structure corresponding to Figure 2a with LP and HP layers having, respectively, porosities *p_LP_* of 60% (corresponding to refractive index *n_LP_* = 2.048) and a *p_HP_* of 80% (corresponding to refractive index *n_HP_* = 1.367), is reported in Figure 2b. Its maximum value depends on the refractive index of each layer and on the number N of the patterns according to:

(6) $$R=\frac{{\left[{\left(\frac{n_{HP}}{n_{LP}}\right)}^{2N}-1\right]}^{2}}{{\left[{\left(\frac{n_{HP}}{n_{LP}}\right)}^{2N}+1\right]}^{2}},$$

### 2.2. Microcavity Theory

**Figure 3:**
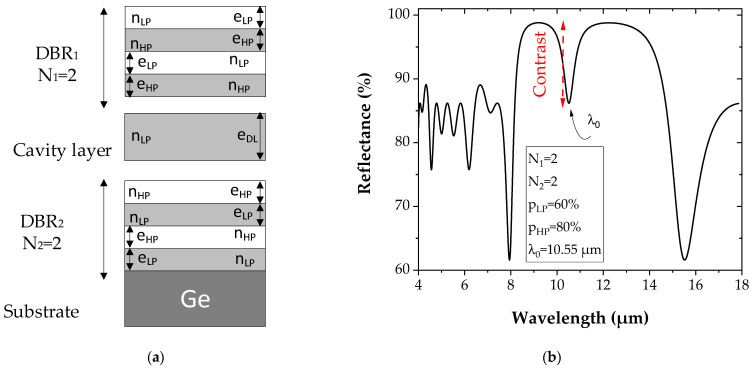
(**a**) Schematic of a PGe microcavity reflector constituted of a Fabry–Perot layer cavity of low porosity (refractive index *n_LP_* and thickness *e_DL_*) sandwiched by two DBRs. The two DBRs are made of a pattern of low (60%) and high porosity (80%) layers with a refractive index *n_LP_* and *n_HP_* and a thickness *e_LP_* and *e_HP_*, respectively, and repeated in this case two times. (**b**) Calculated reflectance spectrum of the PGe microcavity reflector with values of 60% and 80% for the low and the high porosities, respectively.

PGe planar microcavities have also been simulated. The microcavity is constituted of two distributed Bragg reflectors (DBRs) with a Fabry–Perot cavity thickness $e_{DL}$ of λ_0_/2 in the middle (Figure 3a). Several alternating patterns of PGe layers of different refractive indices, repeated N times, constitute the DBRs. For the DBRs, the optical thickness of each single-layer equals *λ*_0_/4, according to the following relation:

(7) $$e_{LP}n_{LP}=e_{HP}n_{HP}=\frac{\lambda_{0}}{4},$$

In the case of a microcavity, the relation becomes:

(8) $$e_{DL}n_{LP}=\frac{\lambda_{0}}{2},$$

A typical reflectance spectrum of a microcavity (Figure 3b) is characterized by a sharp dip in the stop band of the DBR, corresponding to the resonant wavelength λ_0_ of the cavity. This difference between the reflectance level represents the contrast, which is defined by the difference between the maximum (R_max_ = 99%) and minimum (R_min_ = 86%) of the reflectance.

The resonance properties of the microcavity is characterized by the quality factor of the structure. In order to study this parameter, the classical Fabry–Perot can be used [38]. The quality factor (*Q*) is thus given by the following equation:

(9) $$Q=\frac{\lambda }{\Delta \lambda }=\frac{2n_{0}e_{DL}\pi \sqrt{R}\exp(-Ae_{DL}/2)}{\lambda_{0}(1-R\exp(-Ae_{DL}))},$$

where *n*_0_ is the refractive index of the resonant cavity layer that separates the two Bragg mirrors, $e_{DL}$, the thickness of the cavity layer, *λ*_0_ is the expected resonant wavelength equal to 10.55 µm, *A* is the absorption of the molecule, and *R* is the reflectance value.

## 3. Results and Discussion

### 3.1. Bragg Reflector

The objective of this part is to study the influence of two parameters on reflectance: the contrast between the high and low porosities of the multilayers and the number N of layers. The upper layer of the period is the one which has the higher porosity.

**Figure 4:**
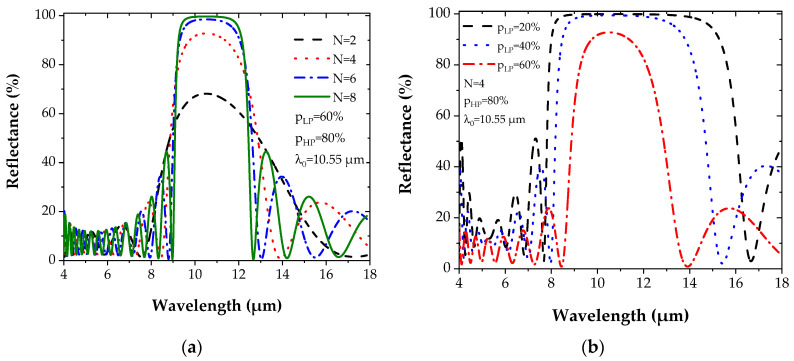
(**a**) The Bragg reflector for PGe materials with (**a**) porosities *p_LP_* = 60% and *p_HP_* = 80% while varying N. (**b**) Reflectance spectrum when N is fixed to 4, porosities *p_HP_* = 80%, and varying *p_LP_*.

Two structures are proposed. The first structure is composed of pairs of layers whose high porosity is *p_HP_* = 80%, while the low porosity is *p_LP_* = 60% and the number N of layers is varied to study its impact on the reflectance evolution (Figure 4a). For the second structure, the number N of layers is fixed to four, while the low porosity value *p_LP_* varies from 20% to 60%, whereas *p_HP_* is constant and equal to 80% (Figure 4b). To keep the optical thickness of each layer constant and have a central wavelength λ_0_ at 10.55 µm (Equation (7)) in all simulations, the thicknesses and the refractive indices are chosen according to Table 1:

Table 1 shows that the layer thickness e increases with the layer porosity.

Thus, *p_HP_* is set to 80%, and the Bragg reflector total thickness is smaller for lower *p_LP_*, and also for a high porosity contrast Δ*p* between the two pattern layers. These results also highlight the interest in Ge which, in addition to having a wider transparency, offers much lower layer thicknesses than low refractive indices materials [34].

Figure 4 represents the evolution of the reflectance spectrum for the Bragg mirror by varying either the number N of layers or the degree of porosity *p_LP_* (*p_HP_* fixed at 80%) and by taking into account the Ge complex refractive index dispersion [35].

The variation of the number N of layers (2 to 8) induces an increase of the maximum reflectance *R*_max_, which approaches 100% for N = 8 while narrowing the band (Figure 4a). The same observation is noticed with the increase of the contrast between the two porosities *p_LP_* and *p_HP_*, but with a widening of the band (Figure 4b).

From Figure 4a, it can also be noticed that a maximum reflectance (100%) is quickly reached with a small number of layers when the contrast Δ*p* between the two porosities *p_HP_* and *p_LP_* is high. These results show the enormous potential of PGe for the fabrication of optical devices based on multilayer structures. Compared to multilayers based on other materials, the maximum reflectance was achieved for a much lower number of layers [34,39,40].

### 3.2. Microcavity

#### 3.2.1. Optimization of the Structure

The microcavities are made up of Bragg reflected mirrors studied in the previous section and of a resonant cavity centered at 10.55 µm for SF_6_ gas detection.

**Figure 5:**
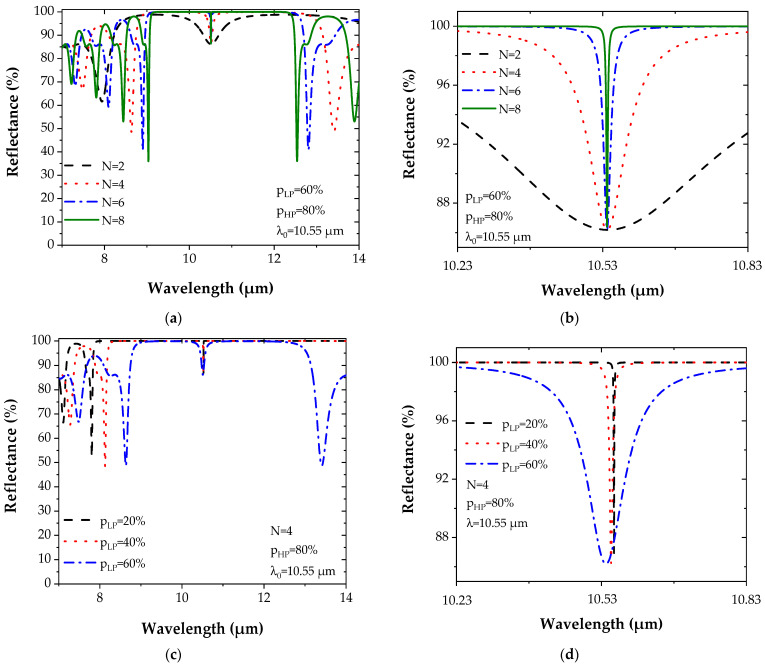
Reflectance spectrum of microcavities based on symmetric PGe Bragg mirrors with: (**a**,**b**) porosities *p_LP_* = 60% and *p_HP_* = 80% while varying N; (**c**,**d**) N fixed to 4, porosities *p_HP_* = 80% and varying *p_LP_*; (**d**) the magnification of the spectra around the resonance wavelength.

Figure 5 reports the reflectance spectrum for symmetric Bragg mirrors with different values of N with N = N_1_ = N_2_, where N_1_ and N_2_ the number of layers of the upper and lower Bragg mirrors, respectively. The increase of the number N of layers allowed the increase of the maximum reflectance R (Figure 5a,b). A reflectance of 100% was reached for N = 4 (Figure 5a,b). The decrease in Δp is accompanied by a narrowing of the band Δλ (Figure 5c,d). The contrast is about 14% and is not influenced by the variation of these previously mentioned parameters (Figure 5c,d).

**Figure 6:**
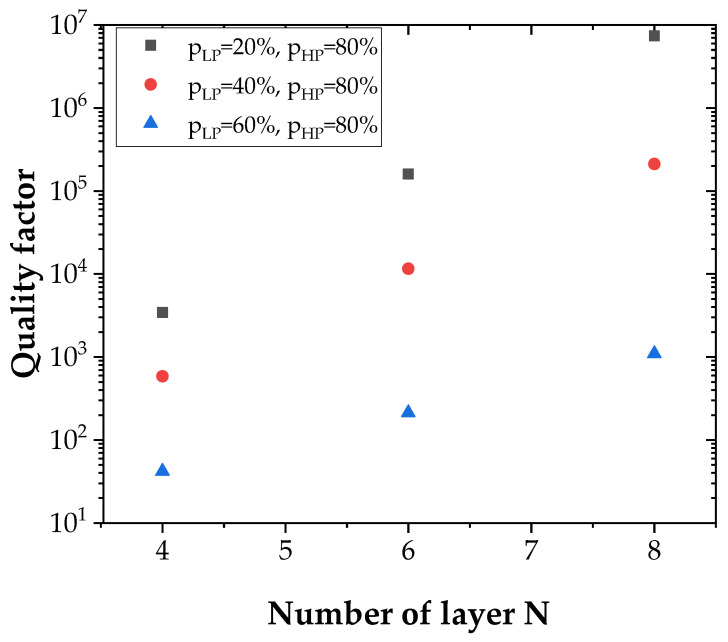
Quality factor Q as a function of layer number N for the microcavities studied.

Equation (9) was used to calculate the quality factor Q. The results, for different configurations, are shown in Figure 6.

If the increase of the number of layers N allows a slight increase of the quality factor Q, values are multiplied by several orders of magnitude when increasing Δp increases. The influence of Δp on the Q factor is therefore larger than that of the number of layers N. The maximum value of Q is reached for N = 8, *p_LP_* = 20% and *p_HP_* = 80%, and is of the order of 7.39 × 10^6^.

In symmetrical microcavities, the narrow peak of Figure 5a–d, which is centered at the wavelength of 10.55 µm, does not go down sufficiently (low contrast), which could limit the detection dynamics. This is due to the strong reflectivity of the top Bragg mirror.

In order to improve the resonance contrast, an asymmetric microcavity composed of two Bragg mirrors whose upper part is less reflective than the lower part has also been studied. To obtain a reflectance difference of between the two DBRs, the number of layers N_1_ (for the upper DBR) and N_2_ for the lower DBR are no longer chosen to be equal.

**Figure 7:**
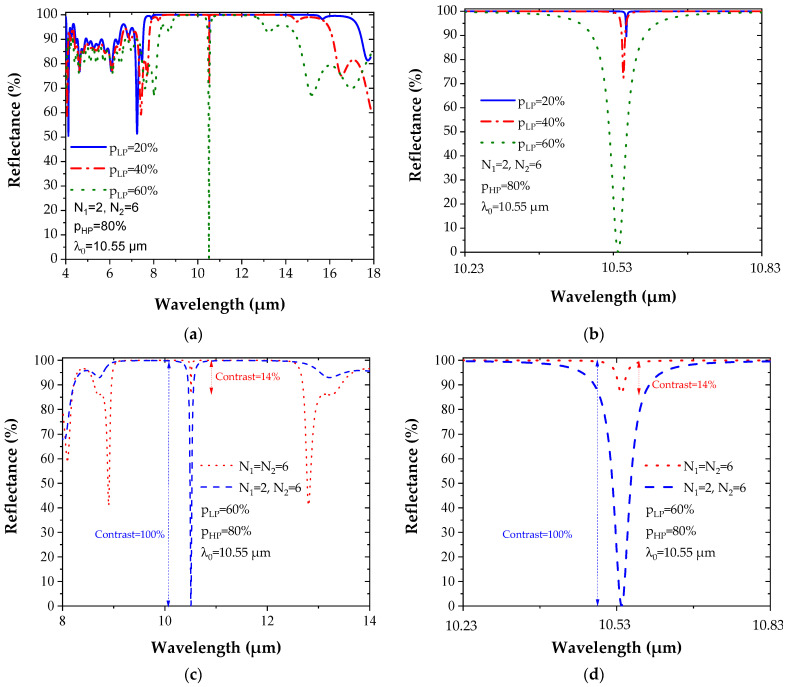
(**a**,**b**) Reflectance spectrum of asymmetric microcavities: N_1_ = 2 and N_2_ = 6, porosities *p_HP_* = 80% and varying *p_LP_*. (**c**,**d**) Comparison between the reflectance spectrum of the symmetric (N_1_ = N_2_ = 6) and asymmetric microcavities (N_1_ = 2 and N_2_ = 6) for *p_HP_* = 80% and *p_LP_* = 60%.

Figure 7a,b shows the reflectance spectrum of an asymmetric microcavity varying Δp and using N_1_ = 2 and N_2_ = 6. The contrast of reflectance is maximum (100%) for *p_LP_* = 60% and *p_HP_* = 80%. Figure 7c,d shows a comparison between the reflectance spectrum of the symmetrical in red (contrast = 14%) and asymmetrical microcavities in blue (contrast = 100%). The asymmetric structure associated with the choice of porosities of *p_LP_* = 60% and *p_HP_* = 80% gives a better contrast. That is the reason why it is used in the next part for SF_6_ gas detection.

#### 3.2.2. Theoretical SF_6_ Gas Detection

The asymmetric microcavity structure used has the following parameters: *p_LP_* = 60%, *p_HP_* = 80% for a respective number of layers on both sides, N_1_ = 2 and N_2_ = 6.

**Figure 8:**
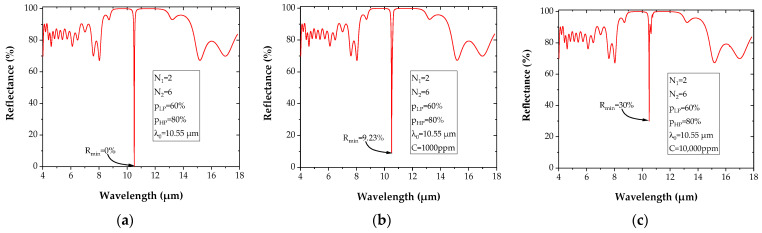
Reflectance spectrum of asymmetric microcavity, for PGe materials: without gas (**a**) and with SF_6_ concentrations in air of (**b**) 1000 ppm, and (**c**) 10,000 ppm.

The chosen asymmetric microcavity is first tested in the absence of gas molecules (n_sup_ = n_air_ = 1). In the presence of SF_6_ gas, the imaginary part k in the superstrate (Equation (2)) is included in the simulation. The SF_6_ concentration C increase leads to an increase of the reflectance R_min_, which corresponds to the lowest reflectance level at resonance. R_min_ is null when there is no gas molecule (Figure 8a) while it reaches about 9% for a SF_6_ concentration of 1000 ppm (Figure 8b), and 30% when C = 10,000 ppm (Figure 8c).

**Figure 9:**
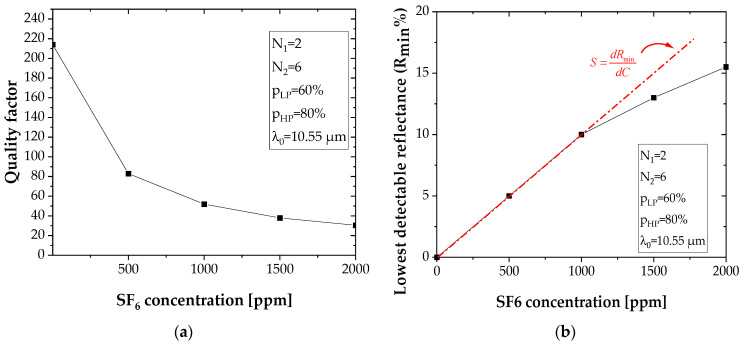
(**a**) Quality factor Q as a function of SF_6_ concentration. (**b**) The lowest detectable reflectance as a function of concentration of target molecules.

A degradation of the quality factor Q is observed as the concentration C of the gas to be detected becomes important (Figure 9a). The opposite phenomenon is observed for the lowest detectable reflectance R_min_ (Figure 9b).

The sensitivity $S=\frac{dR_{\min}}{dC}$, which is assimilated to the tangent to the curve for SF_6_ concentrations inferior to C = 1000 ppm in air (threshold value), is equal to S = 0.0093 ± 0.0014%/ppm.

The limit of detection (LOD), which is the minimum SF_6_ concentration detectable in air is defined by:

(10) $$LOD=\frac{{\left(\Delta R_{\min}\right)}_{\min}}{S},$$

where (ΔR_min_)_min_ is the minimal reflectance variation measurement. Taking into consideration a typical resolution using attenuated total reflectance Fourier transform infrared spectroscopy of 0.00001% [41], a detection limit of 1.0 ± 0.2 ppb could be theoretically estimated, on the assumption of the linear dependence of R_min_ on gas concentration, for the detection of low concentration of SF_6_ under 1000 ppm. This is a promising value since it is 1000 times larger than the state of the art value of 1 ppm obtained by Yingang et al. [31].

## 4. Conclusions

This work presents the use of PGe in multilayers for the SF_6_ mid-IR detection in mid-IR. First, the study focused on the influence of two parameters (number N of layers and the degree of Ge porosity) and on the reflectance R in a structure based on the Bragg mirror. A reflectance of 100% was reached for a number of layers, N = 4, for degrees of porosity *p_LP_* = 20% *p_HP_* = 80%. Using a microcavity consisting of Bragg mirrors previously optimized, a 100% reflectance and a high-quality factor Q = 7.39 × 10^6^ were obtained for a number of symmetrical Bragg mirrors, N_1_ = N_2_ = 8. However, to improve the 14% contrast, an asymmetrical microcavity, consisting of a less reflective top DBR with N_1_ = 2 (68%) and a more reflective bottom DBR with N_2_ = 6 (99.7%), is designed. In these conditions, a contrast of 100% is achieved, which would enable a greater detection dynamic at the resonant wavelength of the microcavity corresponding to an SF_6_ absorption wavelength to insure the sensing selectivity. Theoretical sensing performances of this asymmetrical structure were numerically evaluated for SF_6_ gas detection, and a sensitivity of the order of S = 0.01%/ppm with an estimated LOD of =1 ppb was inferred, outperforming SF_6_ sensing detection limits reported in the literature [31]. As asymmetrical PGe vertical structures are quite easy to elaborate and their use in sensing provide a very rapid response, PGe is a very promising material to be explored for the implementation of multilayer optical sensors for Mid-IR gas detection.

## Figures and Tables

**Table 1:** The calculation of the thickness e and the refractive index n for different values of porosity *p* to obtain λ_0_/4 with λ_0_ = 10.55 µm.

*p* (%)	20	40	60	80
n	3.549	2.844	2.048	1.367
e (nm)	743	927	1288	1929

## Data Availability

Not applicable.
